# Chronic administration of a cannabis-derived mixture at an antihyperalgesic dose does not significantly enhance hepatotoxicity or the development of metabolic dysfunction-associated steatohepatitis in male mice

**DOI:** 10.3389/ebm.2025.10356

**Published:** 2025-05-19

**Authors:** Kim B. Pedersen, Tomislav Jelesijevic, Tamara M. Morris, Sarah M. Melton, Ashley S. Henderson, John F. Glenn, Gregory J. Davenport, Martin J. J. Ronis, Peter J. Winsauer

**Affiliations:** ^1^ Department of Pharmacology and Experimental Therapeutics, Louisiana State University Health Sciences Center, New Orleans, LA, United States; ^2^ Department of Comparative Biomedical Sciences, Louisiana State University School of Veterinary Medicine, Baton Rouge, LA, United States; ^3^ MilMed R&D Consulting LLC, Brunswick, MD, United States; ^4^ Full Spectrum Omega Inc., Los Angeles, CA, United States

**Keywords:** cannabis, liver, Western diet, steatosis, metabolic dysfunction-associated steatohepatitis, MASH

## Abstract

Cannabis and cannabinoid mixtures have been linked to a variety of health benefits including pain mitigation, suppression of nausea produced by chemotherapeutic agents, anti-inflammatory effects, and effects on energy homeostasis, glucose, and lipid metabolism. The latter properties have led to the suggestion that these products could have therapeutic effects on the development of metabolic dysfunction-associated steatohepatitis (MASH) - a severe type of liver pathology in obese and diabetic patients. However, varying agonist and antagonistic properties of different cannabinoids on the endogenous cannabinoid system make prediction regarding hepatic effects and diet interactions difficult. The current study was designed to examine hepatic pathology following chronic administration of a cannabinoid mixture (NEPE14) at a dose equivalent to one previously demonstrating antihyperalgesic effects in rats. The effects of NEPE14 were investigated in a mouse model of MASH produced by feeding a Western diet rich in fat and simple sugars. After 24 weeks of NEPE14 administration, there was no hepatotoxicity in mice receiving the control diet and no significant exacerbation of MASH in mice receiving the Western diet. In conclusion, no chronic liver toxicity was observed, but there was also no evidence for protection against MASH by this product.

## Impact statement

Cannabinoid preparations are increasingly being used for medicinal purposes. Different cannabinoids were previously reported to promote or hinder steatosis of the liver. We evaluated a Non-Euphoric Phytocannabinoid Elixir #14 (NEPE14) at an antihyperalgesic dose for effects on liver pathology and on the development of metabolic dysfunction-associated steatohepatitis (MASH) in male mice. Chronic administration of NEPE14 caused neither amelioration nor worsening of hepatic toxicity, steatosis or inflammation in mice fed a Western diet for 24 weeks.

## Introduction

Medical and recreational use of cannabis and cannabinoid products have increased dramatically as a result of legalization in many states in the US. The most prominent and well characterized phytochemicals in cannabis are delta-9-tetrahydrocannabinol (THC) and cannabidiol (CBD). However, cannabis contains many other potentially bioactive cannabinoids, alkaloids, flavonoids, and terpenoids [1]. The endocannabinoid system, involving signaling through the arachidonic acid-derived ligands anandamide and 2-arachidonylglycerol and cannabinoid receptors 1 and 2 (CB1 and CB2 encoded by genes *CNR1* and *CNR2*) are involved in the regulation of many neuronal systems, immune responses, and energy balance [2–4]. However, research into the potential beneficial or harmful effects of exogenous cannabinoids and other cannabis components has historically been limited until recently due to their registration as Schedule 1 substances by the Drug Enforcement Administration (DEA). Although they remain Schedule 1 substances to date, various state legally-approved medical marijuana programs (MMPs) have allowed for increased focus on these substances’ medical uses. For instance, in addition to the known psychopharmacological and behavioral effects of cannabis, there is evidence from pre-clinical studies and clinical trials that various formulations with individual or multiple cannabis constituents can have anti-inflammatory effects on inflammatory bowel disease, potential anti-obesity effects, and possible protective effects against fatty liver disease [4].

Regarding the latter, the epidemiological and animal studies are inconclusive [4–6]. Both CB1 and CB2 receptors are expressed in liver and have a variety of effects on lipid and glucose homeostasis, insulin sensitivity and development of hepatic steatosis [4, 7]. In general, CB1 agonists induce steatosis via increases in *de novo* fatty acid synthesis and increased expression of lipogenic enzymes, whereas CB2 agonists increase steatosis by increasing expression of CB1 receptors [4]. Cannabinoid receptor antagonists are antisteatotic [4, 5]. Given that THC is a partial agonist it could be predicted to be steatotic. Conversely, CBD can decrease CB1 activation and is anti-inflammatory, which would suggest it to be both anti-steatotic and protective against fatty liver disease [4]. However, predictions about the effects of cannabis and cannabis-derived mixtures on steatosis and progression of fatty liver disease are complicated by the entourage effects of the many different cannabinoids and other phytochemicals in cannabis, and by the potential development of tolerance after chronic use [4–6]. Several studies have observed reduced steatosis in cannabis users [5] and a cross-sectional study found a 43% reduction in the incidence of non-alcoholic steatohepatitis (NASH) in chronic cannabis users [4]. These studies contrast with other studies that have observed no significant effects [5, 7]. Pre-clinical studies with CBD and hemp seed oil have demonstrated anti-steatotic, anti-inflammatory, and antioxidant effects in liver models of Non-Alcoholic Fatty Liver Disease (NAFLD) [8–10]. Phase 2 randomized control trials with CBD showed no significant benefit (Jazz Pharmaceuticals. A Randomized, Partially-blind, Placebo-controlled, Pilot, Dose-ranging Study To Assess The Effect Of Cannabidiol (CBD) On Liver Fat Levels In Subjects With Fatty Liver Disease. 2018.^1^).

NAFLD is a common and increasingly prevalent metabolic disorder [11]. The Western style dietary pattern with fast-food consumption is associated with increased frequency liver steatosis and liver disease [12, 13]. In light of the inconsistent data in the current literature and possible entourage effects of cannabinoids other than THC and CBD [1], the current study was designed to determine the chronic effects of a proprietary cannabis-derived mixture, Non-Euphoric Phytocannabinoid Elixir #14 (NEPE14), at an antihyperalgesic dose on liver pathology [14] and on development of metabolic dysfunction-associated steatohepatitis (MASH) in response to feeding a Western diet.

## Materials and methods

### NEPE 14

Non-Euphoric Phytocannabinoid Elixir #14 (NEPE14) is a proprietary cannabis-derived whole-plant botanical formulation supplied by Full Spectrum Omega Therapeutics, Inc. (Adelanto, CA). It is a mixture of extracts from the Cannabis sativa plant, hemp (hempseed oil) and marijuana (proprietary extract process), and the final product contains less than 0.3% Δ^9^–THC. The concentrations of the major phytocannabinoids are shown in Table 1. A certificate of analysis (COA) with the levels shown in Table 1 was provided for NEPE14 by SC Laboratories California LLC (Santa Cruz, CA). The regulatory compliance testing as reflected in the COA showed no measurable levels of pesticides, mycotoxins, microbes, residual solvents, or heavy metals.

**TABLE 1:** Weight-to-volume assessments for specific cannabinoids identified in the batch of NEPE14 used for this experiment.

Non-euphoric phytocannabinoid elixir 14 (NEPE14)		
Batch #009		
Constituent cannabinoids	mg/mL	LOD/LOQ (mg/mL)
Δ9-THC	0.174	0.0001/0.0005
THCA	0.020	0.0001/0.0002
CBD	0.004	0.0001/0.0004
CBDA	0.016	0.0001/0.001
CBC	0.002	0.0001/0.0004
CBCA	0.0015	0.0001/0.0006
Δ8-THC	0.001	0.0003/0.0008
THCV	0.001	0.0001/0.0004
CBG	0.001	0.0001/0.0002
CBGA	0.001	0.0001/0.0003
CBN	0.001	0.0001/0.0003

The cannabinoids listed were identified by high performance liquid chromatography with diode-array detection (HPLC-DAD). Limit of Detection (LOD) refers to the lowest amount detectable, where Limit of Quantification (LOQ) refers to the lowest amount of an analyte in a sample that can be determined quantitatively with suitable precision/accuracy. The “A” after a cannabinoid, such as THCA, refers to the acid form of the cannabinoid.

### Animals

Male C57Bl/6J mice were purchased from the Jackson Laboratory and housed two per cage. At 16 weeks of age, cages were randomized to receive a high-fat Western diet or a low-fat control diet *ad libitum*. The Western diet was AIN-76A (no. 5342 from TestDiet (Richmond, IN) supplemented with 21.3 g/L fructose and 18.9 g/L glucose in the drinking water, a diet previously developed by Krishnan et al. to produce fibrosing MASH with high fidelity to the human condition [15]. The control diet was AIN-93G (no. 5TJS) from TestDiet with normal drinking water. For each diet, mice in half the cages received NEPE14 and mice in the other half received corn oil provided daily at a volume of 1 μL/g body weight into the cheek pouch at 1,500 ± 90 min providing 0.23 µg of major cannabinoids per g body weight, of which 78% is Δ^9^–THC. This dose of NEPE14 was similar to the lowest antihyperalgesic volume used in a previous study [14]. There were 10 mice per experimental group. At 40 weeks of age, mice were sacrificed by CO_2_ asphyxiation after an overnight fast with collection of serum and tissues. The Institutional Animal Care and Use Committee (IACUC) of LSU Health Sciences Center approved the animal experiments.

### Serum parameters

Alanine transaminase (ALT), aspartate aminotransferase (AST), triglycerides, cholesterol and insulin were determined by kits ab105134 (Abcam), MET-5127 (Cell Biolabs, Inc.), #10010303 (Cayman), #10007640 (Cayman) and ab277390 (Abcam), respectively. Glucose was measured with kit ab65333 (Abcam) in serum after deproteinization with 10 kDa spin columns ab93349 (Abcam). HOMA-IR was calculated from the fasted insulin and glucose levels.

### Liver pathology

The left liver lobe was fixed for 3 days in alcoholic formalin made of 0.2 L 37% formaldehyde, 1.2 L 100% ethanol and 0.6 L H_2_O followed by storage in 10% neutral buffered formalin. H&E-stained slides were scored by a board-certified veterinary anatomic pathologist blinded to the treatment groups for steatosis and inflammatory foci. Microsteatosis and macrosteatosis were graded separately for the periportal, midzonal and centrilobular zones with scores of 0, 1, 2 and 3 if steatosis was present in <5%, 5%–33%, 33%–66% and >66%, respectively, of the hepatocytes. Overall microsteatosis and macrosteatosis scores were the averages for the three zones. The total steatosis score was the average of the microsteatosis and macrosteatosis scores. Inflammatory foci were counted in six fields with a ×10 objective. Fibrosis was assessed after picrosirius red (PSR) staining. PSR stains collagen red and most other cellular components yellow. Liver parenchyma PSR (red) staining was quantified by the same ImageJ settings (threshold 50 for green image in a RGB stack) from four images from each mouse. The images were acquired by an Olympus BX43 microscope with DP28 camera. Liver triglycerides were determined from 87.5 to 100 mg flash-frozen liver tissue using the kit #10010303 (Cayman) and a TissueLyser II instrument (Qiagen, Hilden, Germany).

### Immunohistochemistry

Five-micrometer-thick tissue liver sections were deparaffinized and rehydrated. Heat-mediated antigen retrieval was achieved in a pressure cooker at a temperature of 115–118°C and a pressure of 10.2–11.6 psi for 10 min. After blocking with Invitrogen eBioscience IHC/ICC blocking buffer–high protein (Fisher Scientific #50-184-85), slides were incubated with rabbit anti-CD3e (Fisher Scientific #PIMA535204) at a 1:1,000 dilution or rat anti-CD19 (Life Technologies #14-0194-80) at a 1:300 dilution primary antibodies. Alkaline phosphatase-conjugated goat anti-rabbit and rabbit anti-rat antibodies (Fisher Scientific #A18874 and # A18918, respectively) were added to the slides at a 1:500 dilution. Slides were incubated with a Vulcan Fast Red Chromagen kit (Fisher Scientific #50-828-59) for 10 min, washed with distilled water and counterstained with Harris hematoxylin (ThermoFisher Scientific #1859352). Slides were dehydrated and cover-slipped with Mounting Medium (ThermoFisher Scientific #1859351). The images were acquired using an Olympus BX43 microscope with an Olympus DP28 color camera.

### QRT-PCR

The left part of the medial liver lobe was immersed in RNAProtect overnight at 4°C, followed by storage at −80°C until RNA extraction. RNA was extracted from 20 to 30 mg liver tissue using a TissueLyser II instrument and an RNAeasy Plus Mini Kit (Qiagen). RNA concentration was determined by absorbance at 260 nm on a NanoDrop spectrophotometer, and the quality assessed on a TapeStation 2200 instrument (Agilent Technologies, Santa Clara, CA). All RNA extracts were of high quality with RNA Integrity Numbers (RIN) between 8.9 and 9.6. Assays were run with Power SYBR Green RNA-to-CT 1-step kit reagents (Thermo Fisher Scientific, Waltham, MA) on a LightCycler 480 II (Roche, Indianapolis, IN). Primers are listed in Table 2. Targets were normalized to the amount of total RNA with each reaction containing 0.5 ng RNA for 18S rRNA and 5 ng RNA for all other targets.

**TABLE 2:** qRT-PCR primers.

Target	Gene	Forward primer	Reverse primer
18S rRNA		CGG​ACA​GGA​TTG​ACA​GAT​TG	CAA​ATC​GCT​CCA​CCA​ACT​AA
COL1A1	*Col1a1*	CTG​ACG​CAT​GGC​CAA​GAA​GA	ATA​CCT​CGG​GTT​TCC​ACG​TC
αSMA	*Acta2*	GTG​GCT​ATT​CCT​TCG​TGA​CTA​C	GAG​CTA​CAT​AGC​ACA​GCT​TCT​C
TNFα	*Tnf*	TTC​TAT​GGC​CCA​GAC​CCT​CA	CAC​TTG​GTG​GTT​TGC​TAC​GA
IL-6	*Il6*	CTT​CAC​AAG​TCG​GAG​GCT​TAA​T	GCA​AGT​GCA​TCA​TCG​TTG​TTC
IFN-γ	*Ifng*	ATC​GGC​TGA​CCT​AGA​GAA​GA	AGC​CAA​GAT​GCA​GTG​TGT​AG
B220	*Ptprc*	CCC​TTC​TTC​TGC​CTC​AAA​GT	CAC​CTG​GAT​GAT​ATG​TGG​TCT​C
CD3E	*Cd3e*	GAC​GAT​GCC​GAG​AAC​ATT​GA	GCT​TCT​GAG​GCA​GCT​CTT​G
CYBB	*Cybb*	AGA​GTC​GGG​ATT​TCT​GAC​CG	GCC​CCC​TTC​AGG​GTT​CTT​GAT
CB2	*Cnr2*	GCT​GAC​AAA​TGA​CAC​CCA​GT	AGC​CGT​TGG​TCA​CTT​CTG​TC
CD36	*Cd36*	GCA​AAA​CGA​CTG​CAG​GTC​AA	CAC​CAA​TGG​TCC​CAG​TCT​CA
FATP1	*Fatp1*	GTC​CGC​AAT​GAG​TTC​ACC​CT	GCT​TGA​CGA​CTG​CCT​TGA​CT

### Statistics

When considered appropriate, data were analyzed by an analysis of variance (ANOVA) with comparisons of means with Tukey’s adjustment. For qRT-PCR data, two-way ANOVA was conducted of cycle threshold (C_T_) values with conversion to linear scale for figure presentation. For liver pathology scores, non-parametric Kruskal-Wallis tests were conducted with Dunn’s test for comparison of rank means. Incidence data were analyzed by pair-wise Fisher’s exact test. Effects and differences were considered statistically significant for test probabilities P < 0.05. Statistical analyses were conducted with GraphPad Prism 10.2.0. Unless otherwise stated, figures show means and standard error of means in addition to individual measurements.

## Results

### The Western diet increases body, liver, and fat mass

**FIGURE 1:**
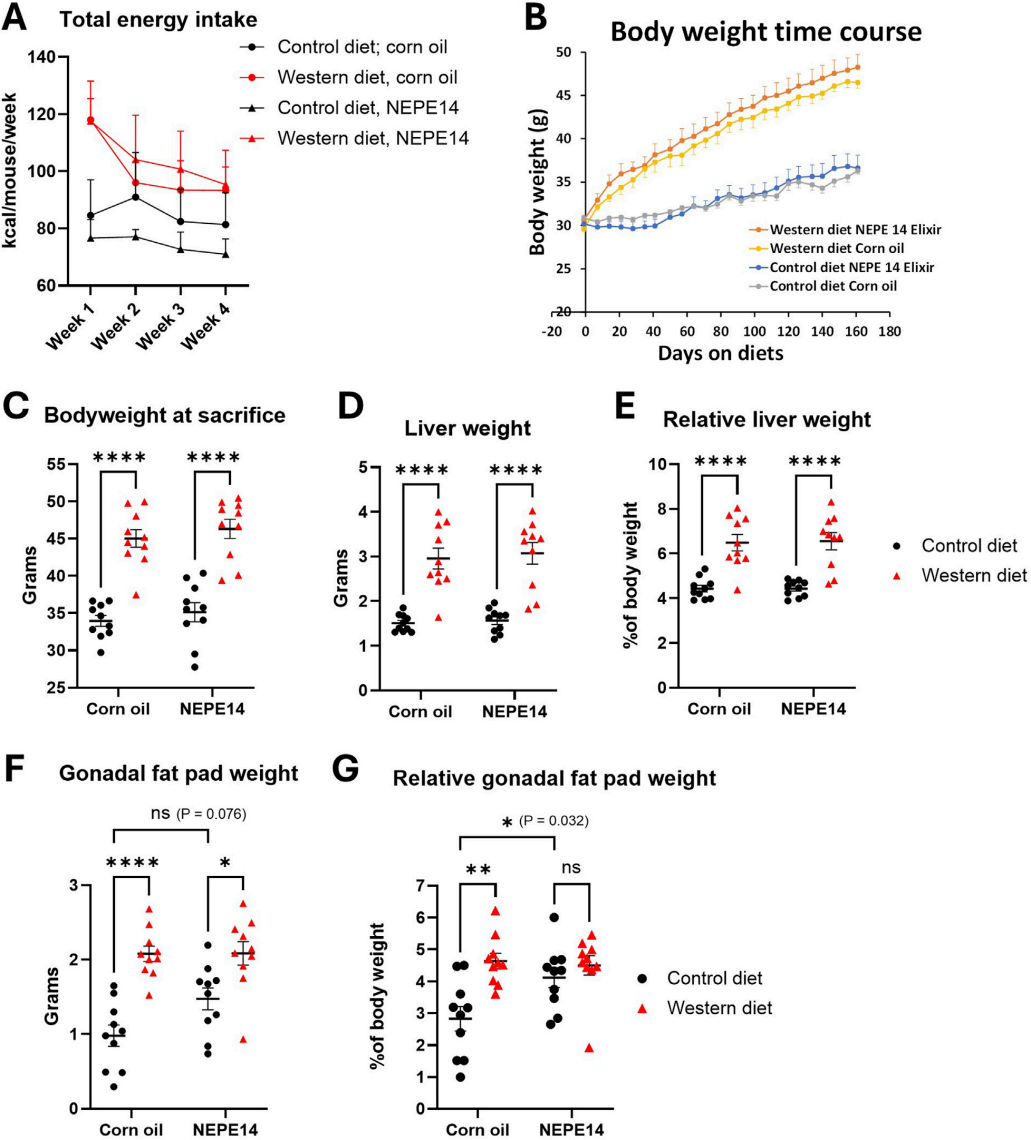
The Western diet promotes obesity. Mice were fed either a control diet or a Western diet and administered daily with corn oil or with NEPE14 for a dosage of 0.23 µg cannabinoids per Gram of body weight. **(A)** The total energy intake from pelleted food, drinking water with glucose and fructose, and corn oil/NEPE14 treatment in the first four weeks of the experiment was calculated. N = 5 cages per group with two mice per cage. **(B)** Time course of body weight; N = 10 mice per group. At sacrifice, body weight **(C)**, liver weight **(D)**, relative liver weight **(E)**, gonadal fat pad weight **(F)** and relative gonadal fat pad weight **(G)** were determined. ns: not significant. *, **, ****: P < 0.05, 0.01, 0.0001 for post-hoc comparisons after two-way ANOVA with Tukey’s adjustment.

The daily dosage of NEPE14 (1 μL/g body weight/day) provided 0.23 µg of quantified cannabinoids per Gram of body weight, of which 78% was Δ^9^–THC (Table 1). The mean intake for each experimental group was monitored for the first four weeks. The average energy intake in kcal was higher for the Western diet than control diet, with no indication that NEPE14 significantly increased intake (Figure 1A). Body weights increased faster for mice fed the Western diet than mice fed the control diet (Figure 1B). At sacrifice, mice given the Western diet had significantly higher body weights than mice given the control diet with no significant effect of the corn oil/NEPE14 administration and no significant interaction between these factors (Figure 1C). With the Western diet, the liver weights and liver weights relative to body weights were significantly increased (Figures 1D,E) with no significant effects of NEPE14. The gonadal fat pad weight was likewise increased for mice fed the Western diet with no significant effect of NEPE14 (Figure 1F). Relative to body weight, NEPE14-treated mice had significantly increased relative gonadal fat pad weight when fed the control diet, but not the Western diet, with a significant (P = 0.032) interaction between diet and NEPE14 administration (Figure 1G).

### The Western diet promotes hyperglycemia, dyslipidemia, and hepatic injury

**FIGURE 2:**
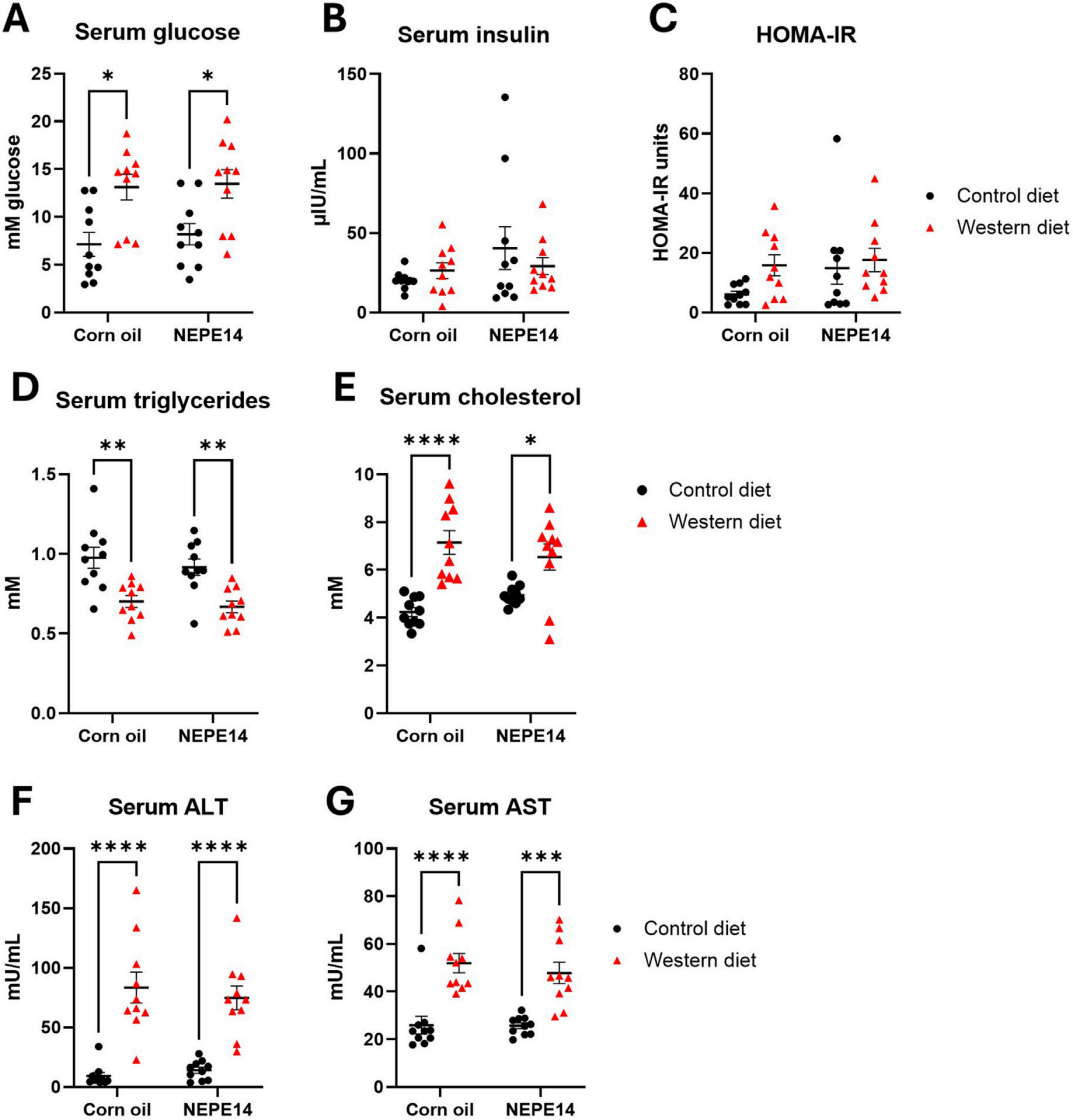
Serum parameters. Blood drawn from mice fasted overnight was used to determine serum glucose **(A)**, insulin **(B)**, HOMA-IR **(C)**, triglycerides **(D)**, cholesterol **(E)**, alanine transaminase (ALT) (**F)**, and aspartate aminotransferase (AST) (**G)**. *, **, ****: P < 0.05, 0.01, 0.0001 for post-hoc comparisons after two-way ANOVA with Tukey’s adjustment.

Mice fed the Western diet had significantly higher fasting glucose than mice fed the control diet (Figure 2A), while there were no significant effects for serum insulin or the HOMA-IR parameter for insulin resistance (Figures 2B,C). The Western diet further led to decreased levels of serum triglycerides and increased levels of total serum cholesterol (Figures 2D,E). Mice fed the Western diet also had significantly increased concentrations of ALT and AST, indicative of liver damage (Figures 2F,G). There were no significant effects of the corn oil/NEPE14 treatment and no significant interactions between diet or NEPE14 administration for any of these serum parameters.

### The Western diet promotes a liver phenotype consistent with metabolic dysfunction-associated steatohepatitis (MASH)

**FIGURE 3:**
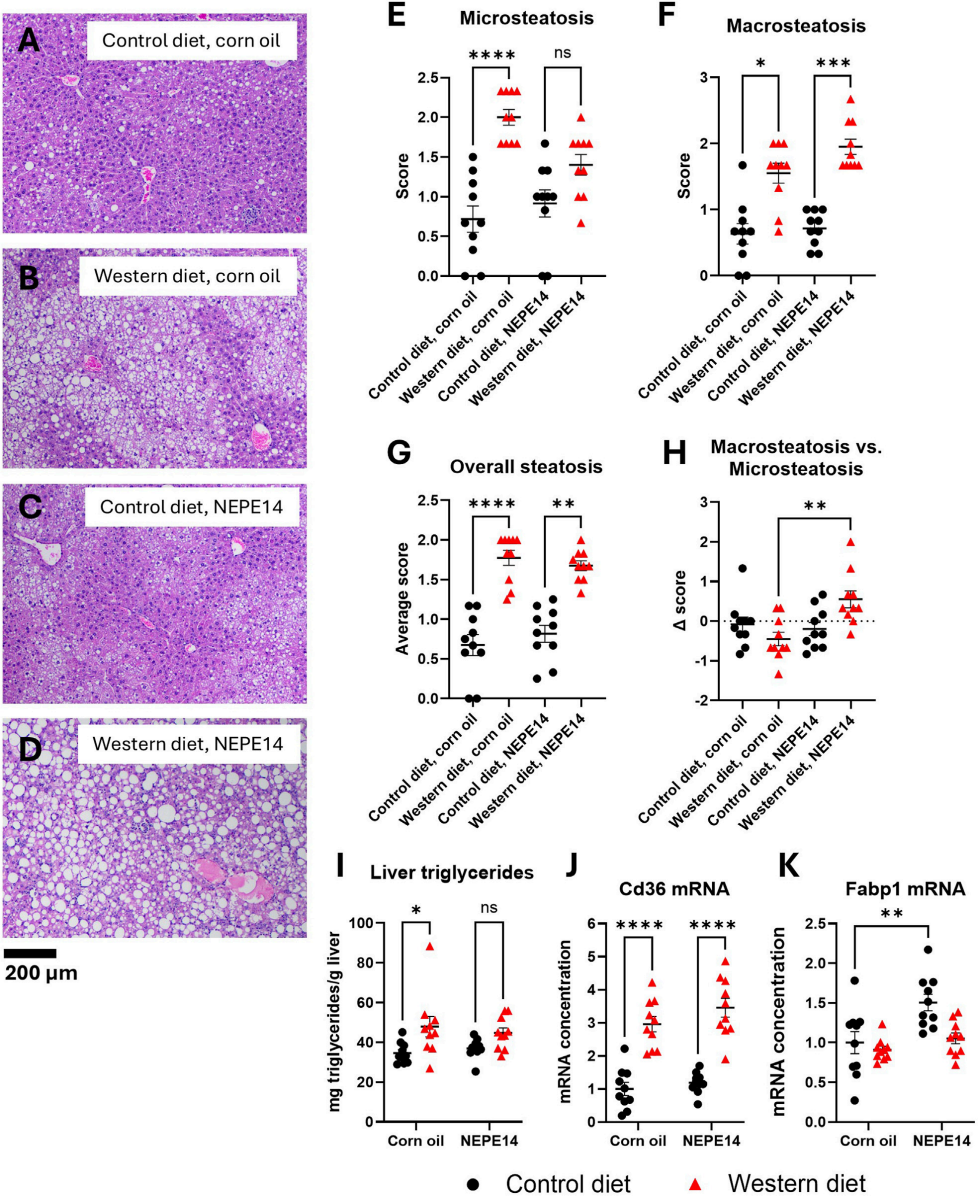
The Western diet promotes liver steatosis. Examples of H&E stained liver sections from each of the four treatment groups **(A–D)**. The livers were scored for microsteatosis **(E)**, macrosteatosis **(F)**, overall steatosis **(G)** and difference (Δ score) in macrosteatosis and microsteatosis scores **(H)**. *, **, ***, ****: P < 0.05, 0.01, 0.001, 0.0001 for post-hoc comparisons after Kruskal-Wallis test with Dunn’s test. **(I)** Liver triglyceride content was determined. ns: not significant. *: P < 0.05 for post-hoc comparisons after two-way ANOVA with Tukey’s adjustment. Hepatic concentrations of Cd36 mRNA **(J)** and Fabp1 mRNA **(K)** were determined and scaled to a value of 1 for the mean of the control diet + corn oil group. **, ****: P < 0.01, 0.0001 for post-hoc comparisons after two-way ANOVA with Tukey’s adjustment.

Livers from mice fed the Western diet tended to show a higher degree of steatosis than livers from mice fed the control diet (Figures 3A–D). The index of microsteatosis was significantly increased by the Western diet in mice treated with corn oil, while an index of macrosteatosis was significantly increased by the Western diet for both treatment groups (Figures 3E,F). The combined score of micro- and macrosteatosis was significantly increased by the Western diet (Figure 3G). To assess the degree of macrosteatosis relative to microsteatosis, we calculated the difference between the macrosteatosis and microsteatosis score (delta score). For mice fed the Western diet, this parameter was significantly increased (P = 0.005) by NEPE14 administration (Figure 3H). We further determined that the amount of liver triglycerides per Gram of liver tissue was significantly increased by the Western diet (P = 0.002 for main effect of diet) (Figure 3I). Thus, during intake of the Western diet, NEPE14 seems to shift storage of hepatic triglycerides towards macrosteatosis without alteration of the total amount of stored triglycerides.

CD36 is a fatty acid translocase with a possible role in pathogenesis of NAFLD. Hepatic expression of CD36 tends to be elevated in conditions of NAFLD, and CD36 overexpression can promote lipogenesis and steatosis [16, 17]. We observed that the concentration of Cd36 mRNA was significantly higher in mice fed the Western diet than in mice fed the control diet with no clear effect of NEPE14 (Figure 3J). Fatty acid binding protein 1 (FATP1) is an abundant hepatocyte protein that is important for intracellular fatty acid trafficking and protection from lipotoxicity of free fatty acids [18, 19]. In at least some cohorts of humans with steatosis and in some mouse models of NAFLD, hepatic expression of FATP1 is diminished. FATP1 also binds cannabinoids, including THC and CBD, which plays a role in hepatic detoxification of cannabinoids [20, 21]. We observed a small, but significant, upregulation of Fabp1 mRNA by NEPE14 administration in mice fed the control diet (Figure 3K).

**FIGURE 4:**
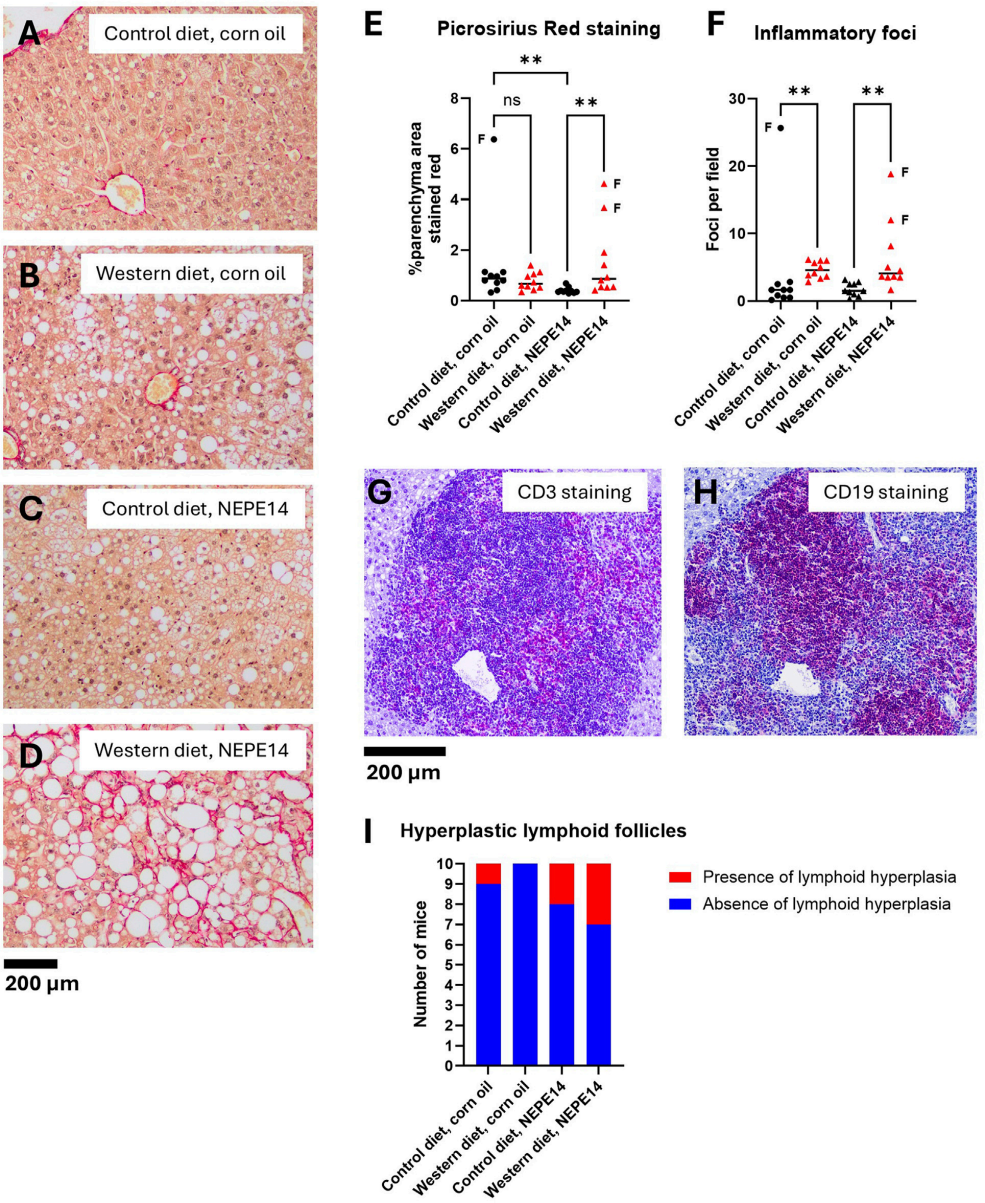
The Western diet promotes liver inflammation. Examples of liver sections stained with picrosirius red from each of the four treatment groups **(A–D)**. The sample in **(D)** was taken from one of the mice with a stiff, fibrotic liver. **(E)** Quantification of parenchymal PSR stained area. **(F)** Counting of inflammatory foci from H&E stained liver sections. In panels **E** and **F**, the horizontal lines indicate medians. The data points indicated by letter “F” are from stiff, fibrotic livers. ns: not significant. **: P < 0.01 for post-hoc comparisons after Kruskal-Wallis test with Dunn’s test. **(G,H)** An example of immunohistochemistry of a hyperplastic lymphoid follicle from a mouse fed the control diet and exposed to NEPE14 with the red-purple color demonstrating the occurrence of both CD3e-positive **(G)** and CD19-positive cells **(H)**. **(I)** Incidence of mice with hyperplastic lymphoid follicles observed in H&E stained liver sections.

Collagen was stained red with picrosirius red (PSR), and the sections were counterstained with hematoxylin. Hematoxylin stains nuclei and their nucleoli, generating a small amount of background signal. An image from each group is presented in Figures 4A–D. At sacrifice, upon macroscopic examination, three mice had stiff, fibrotic livers. Two of these were mice that were fed the Western diet and received NEPE14, while the third was fed the control diet and administered corn oil. The three livers found to be highly fibrotic at sacrifice showed the highest degree of parenchymal PSR staining (Figures 4D,E). The Western diet led to significantly increased staining with picrosirius red in mice administered NEPE14 (P < 0.05). However, this increase was largely due to the two out of ten livers that were highly fibrotic (Figure 4E). In addition, there was a significant decrease in PSR staining produced by NEPE14 administration in mice fed the control diet, even though the staining intensity was already low in nine of the corn oil-administered mice.

The number of foci with inflammatory cells was significantly increased in mice fed the Western diet (Figure 4F). In some of the livers, there were one to three, small to medium size hyperplastic lymphoid follicles containing both CD3 positive (T-lineage cells) and CD19 positive (B-lineage cells) (Figures 4G,H). The incidence was higher in mice administered NEPE14 (Figure 4I), but this was not statistically significant (P = 0.18 by Fisher’s exact test). In addition, there were three incidental findings in mice without hyperplastic lymphoid follicles: 1) a large pancreatic cyst in a mouse that received the Western diet and corn oil; 2) a hyperplastic nodule with a focus of cellular alteration in a mouse with a fibrotic liver that received the Western diet and NEPE14; and 3) a hepatocellular adenoma and moderate bile duct hyperplasia in a mouse with a fibrotic liver that received the control diet and corn oil (data not shown).

**FIGURE 5:**
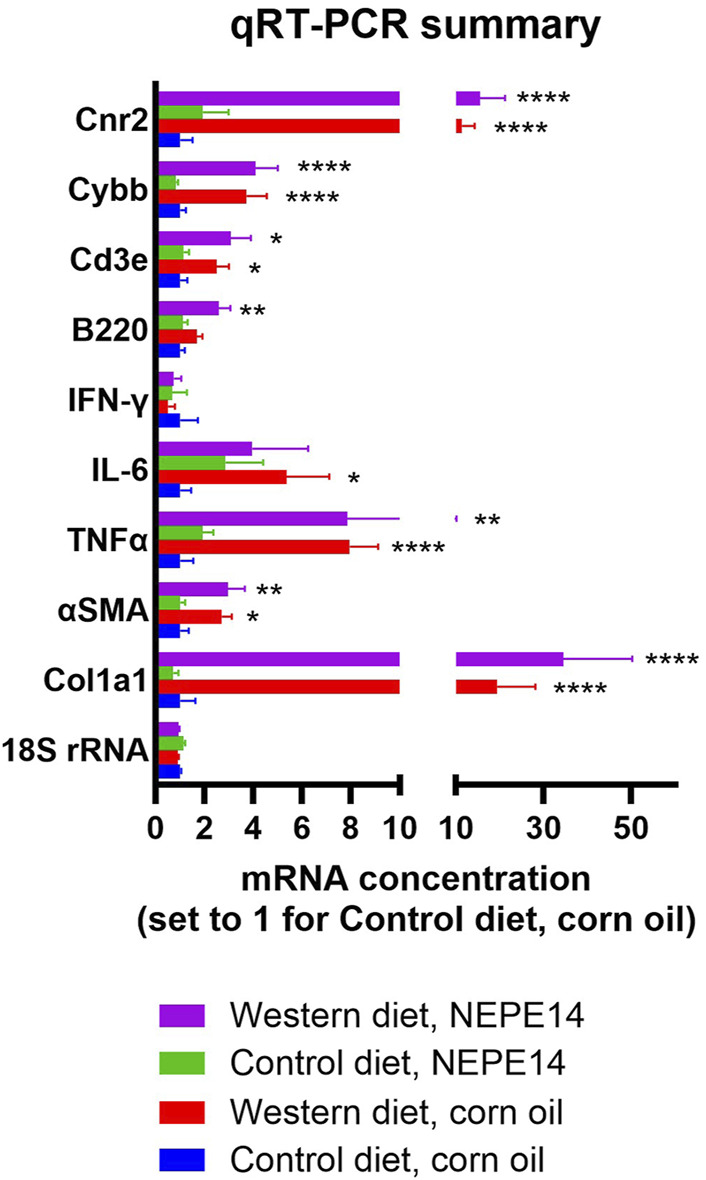
Hepatic mRNA expression. qRT-PCR was conducted for 18S rRNA as a house keeping gene, genes involved in fibrosis (Co1a1 and αSMA), genes involved in inflammation (TNFα, IL-6, IFN-γ, B220, CD3e and Cybb), and the gene encoding cannabinoid receptor 2 (Cnr2). N = 10 mice per experimental group. *, **, ****: P < 0.05, 0.01, 0.0001 for post-hoc comparisons after two-way ANOVA with Tukey’s adjustment.

PSR staining provides a measure of collagen accumulation over time. To get a snapshot of collagen production at the time of sacrifice, we measured expression of mRNA for collagen 1a1 (Col1a1) and for alpha smooth muscle actin (αSMA) which is a marker of stellate cell activation. Concentrations of Col1a1 and αSMA mRNA were increased to the same degree by the Western diet (P < 0.05) in both the control and NEPE14 groups (Figure 5), suggesting activation of stellate cells promoting fibrosis. A key feature of steatohepatitis is enhanced inflammation. In addition to counting inflammatory foci (Figure 4F), we assessed the inflammatory milieu by determining expression of mRNAs for proinflammatory cytokines TNFα, IL-6 and interferon-γ that are upregulated in conditions of steatohepatitis [22–24]. We further measured B220 as a marker of B-cells; CD3e as a marker of T-cells; and the catalytic subunit CYBB of NOX2 that is a marker of Kupffer cells and other phagocytic cells with a role in the immune response. The Western diet led to significantly increased expression of mRNAs for TNFα, IL-6, B220, CD3e and CYBB (P < 0.0001, P = 0.015, P = 0.001, P = 0.0001, and P < 0.0001, respectively, for the main effects of diet) consistent with an enhanced inflammatory state (Figure 5). There were no significant effects of NEPE14 and no significant interactions between diet and chronic NEPE14 administration for any of these parameters. Finally, as receptors for phytocannabinoids, we explored expression of CB1 and CB2 receptors (expressed from genes *Cnr1* and *Cnr2*), and TRPV1 receptors. Likely due to low expression, we could not design robust qRT-PCR assays for Cnr1 and Trpv1 mRNA. Expression of Cnr2 mRNA was induced by the Western diet, with no significant effects of NEPE14 (Figure 5).

## Discussion

In agreement with Krishnan et al. [15], chronic consumption of a Western diet rich in fat and simple sugars resulted in development of obesity and liver pathology consistent with fibrosing metabolic dysfunction-associated steatohepatitis (MASH) in male C57BL/6 mice. Furthermore, serum glucose and cholesterol were elevated in both studies. There were a small number of differences with the current study. We did not observe any significant increase in serum insulin, which may be due to the length of time between removal of the diet and sacrifice, which was 6 h in Krishnan et al. [15] and an overnight fast in the current study. We further observed a significant decrease in serum triglycerides, consistent with other studies of rodents fed a Western diet [25]. Feeding mice with the Western diet also promoted traits of the liver associated with development of MASH such as increased liver weight, increased liver steatosis, increased infiltration of inflammatory cells, and increased expression of profibrotic and proinflammatory genes. NEPE14, a cannabis-derived whole-plant botanical formulation has previously been shown to have significant antihyperalgesic effects when administered either intraperitoneally or at the current dose via the cheek pouch [14]. Given potential entourage effects between the different cannabinoid ingredients and other phytochemicals present in NEPE14, it was difficult to predict what effects chronic administration would have on energy utilization, glucose and lipid homeostasis, and liver pathology. The only significant effect of NEPE14 on body composition was a significant increase in relative adipose tissue weight in mice fed the control diet. This contrasts with previous studies showing weight loss and improved adiposity in animals administered delta-9-THC chronically [4]. For mice fed the control diet, administration of NEPE14 did not cause overt liver pathology, indicating a lack of hepatotoxicity associated with this particular phytocannabinoid mixture. Decreased PSR staining and increased expression of Fatp1 mRNA in mice administered NEPE14 could be beneficial effects, although that would need to be substantiated in future studies. For mice fed the Western diet, no significant differences were observed in the development of overall MASH pathology or gene expression with or without co-administration of NEPE14. However, there were some subtle differences related to the nature of hepatic fat accumulation. A significant shift in lipid droplet size toward macrosteatosis was observed after NEPE14 administration, suggesting a change in lipid packaging, but overall levels of steatosis and triglyceride accumulation were similar between the two groups that received the Western-style diet.

Limitations of the current study include using only male mice and only one volume of NEPE14. Future studies that include female mice and higher doses may be important, as females have been shown to be more sensitive than males to the antinociceptive effects of cannabinoids. In one study, for example, CB1 and CB2 antagonists were used to demonstrate that while both the acute antinociceptive and motoric effects of Δ9-THC were produced via activation of CB1 receptors, the CB1 antagonist rimonabant was up to 10 times more potent in female than male rats and the antagonism of the Δ9-THC-induced antinociception was greater than the antagonism for the motor impairment [26].

The occurrence of stiff, fibrotic livers in three mice, especially for a mouse on the control diet that was administered corn oil, was unexpected and contributes to the likelihood that these findings were idiopathic. Nevertheless, these livers also showed the highest degree of bridging and intralobular fibrosis by picrosirius red staining and the highest number of inflammatory foci. While we did not substantiate mRNA expression data with data on protein levels, it was remarkable that the mice with these livers also had the highest expression of mRNA for COL1A1, TNFα, IFN-γ, CD3E and CYBB within their respective experimental groups (data not shown), demonstrating an enhanced inflammatory state. The focus of cellular alterations found in the fibrotic liver of a mouse that received the Western diet and NEPE14, as well as the hepatocellular adenoma found in the fibrotic liver of a mouse that received the control diet and corn oil, could not be directly tied to a particular diet or substance administered due to the inconsistent occurrence of these pathological findings across the experimental groups. Interestingly, the role of endogenous cannabinoids on hematopoietic cell proliferation, differentiation, and migration has been found to be both positive and negative. For example, most fatty acid ethanolamides such as anandamide inhibit migration, whereas most fatty acid glyceryl esters stimulate migration [27]. The role of the cannabinoid receptors in these effects has also been difficult to establish as experiments with both CB1 and CB2 receptor antagonists indicated that the effects of 2-arachidonyl glycerol on cell migration were receptor dependent, but the effects of anandamide on cell migration were receptor independent.

Hepatic lymphoid hyperplasia was another unexpected finding. In humans, hepatic reactive lymphoid hyperplasia is a rare, but apparently benign condition [28]. While we cannot exclude the possibility of NEPE14 stimulating lymphoid hyperplasia, the incidence in NEPE14-administered mice was not significantly higher than in mice administered the corn oil control. There are reports of NEPE14 increasing lymphocyte counts in response to total body irradiation combined with skin wounding [29]; however, associating this type of bone marrow clonogenicity solely with NEPE administration would be difficult without further studies because NAFLD alone can increase lymphocyte counts [30]. These data would also contrast with data demonstrating that cannabinoids often decrease lymphocyte counts. In one study, for instance, both purified delta-9-tetrahydrocannabinol (THC) and cannabidiol (CBD), along with THC- and CBD-enriched cannabis extracts, reduced activated lymphocyte proliferation in a murine bone marrow transplantation (BMT) model [31]. This study was also able to show that THC, but not CBD, produced this effect through CB2 receptors using splenocytes from CB2 knockout mice in a succinyl ester (CFSE)-labeled lymphocyte proliferation model. Another notable finding from this study was the effect of THC alone on lymphocyte proliferation, as it was eliminated in splenocytes lacking CB2 receptors, but was only less potent after the THC-enriched extract - suggesting that other constituents of the extract might also be responsible for limiting lymphocyte proliferation.

In conclusion, a Western diet for 24 weeks resulted in the development of obesity and liver pathology consistent with fibrosing metabolic dysfunction-associated steatohepatitis (MASH) in male C57BL/6 mice administered either corn oil or the cannabis-derived mixture NEPE14. The relative absence of an effect with NEPE14 in mice fed the Western diet indicated it did not worsen hepatotoxicity at a volume previously shown to have significant antihyperalgesic effects for pain management. It did, however, increase hepatic macrosteatosis compared to microsteatosis over this time period in mice fed the Western diet and produce small increases in relative adiposity in mice fed the control diet. Thus, NEPE14 administration neither significantly reduced nor enhanced development of MASH in response to a Western diet in this mouse model.

## Data Availability

The original contributions presented in the study are included in the article, further inquiries can be directed to the corresponding author.

## Footnotes

1
https://clinicaltrials.gov/study/NCT01284634
